# Chemokine CCL9 Is Upregulated Early in Chronic Kidney Disease and Counteracts Kidney Inflammation and Fibrosis

**DOI:** 10.3390/biomedicines10020420

**Published:** 2022-02-10

**Authors:** Christian Hemmers, Corinna Schulte, Julia Wollenhaupt, Dickson W. L. Wong, Eva Harlacher, Setareh Orth-Alampour, Barbara Mara Klinkhammer, Stephan H. Schirmer, Michael Böhm, Nikolaus Marx, Thimoteus Speer, Peter Boor, Joachim Jankowski, Heidi Noels

**Affiliations:** 1 Institute for Molecular Cardiovascular Research (IMCAR), University Hospital RWTH Aachen, 52074 Aachen, Germany; chemmers@ukaachen.de (C.H.); cschulte@ukaachen.de (C.S.); jwirth@ukaachen.de (J.W.); evstraussfel@ukaachen.de (E.H.); salampourrajabi@ukaachen.de (S.O.-A.); jjankowski@ukaachen.de (J.J.); 2 Institute of Pathology, University Hospital RWTH Aachen, 52074 Aachen, Germany; dwong@ukaachen.de (D.W.L.W.); bklinkhammer@ukaachen.de (B.M.K.); pboor@ukaachen.de (P.B.); 3 Saarland University, 66424 Homburg/Saar, Germany; schirmer@kardiopraxis-schirmer.de (S.H.S.); michael.boehm@uks.eu (M.B.); 4 Department of Internal Medicine I, Cardiology, University Hospital RWTH Aachen, 52074 Aachen, Germany; nmarx@ukaachen.de; 5 Translational Cardio-Renal Medicine, Saarland University, 66424 Homburg/Saar, Germany; timo.speer@uks.eu; 6 Department of Nephrology and Clinical Immunology, University Hospital RWTH Aachen, 52074 Aachen, Germany; 7 Department of Pathology, Cardiovascular Research Institute Maastricht (CARIM), Maastricht University, 6211 LK Maastricht, The Netherlands; 8 Department of Biochemistry, Cardiovascular Research Institute Maastricht (CARIM), Maastricht University, 6211 LK Maastricht, The Netherlands

**Keywords:** chronic kidney disease, chemokine, inflammation, macrophage, CCL6, CCL9, MIP-1γ, fibrosis, collagen

## Abstract

Inflammation and fibrosis play an important pathophysiological role in chronic kidney disease (CKD), with pro-inflammatory mediators and leukocytes promoting organ damage with subsequent fibrosis. Since chemokines are the main regulators of leukocyte chemotaxis and tissue inflammation, we performed systemic chemokine profiling in early CKD in mice. This revealed (C-C motif) ligands 6 and 9 (CCL6 and CCL9) as the most upregulated chemokines, with significantly higher levels of both chemokines in blood (CCL6: 3–4 fold; CCL9: 3–5 fold) as well as kidney as confirmed by Enzyme-linked Immunosorbent Assay (ELISA) in two additional CKD models. Chemokine treatment in a mouse model of early adenine-induced CKD almost completely abolished the CKD-induced infiltration of macrophages and myeloid cells in the kidney without impact on circulating leukocyte numbers. The other way around, especially CCL9-blockade aggravated monocyte and macrophage accumulation in kidney during CKD development, without impact on the ratio of M1-to-M2 macrophages. In parallel, CCL9-blockade raised serum creatinine and urea levels as readouts of kidney dysfunction. It also exacerbated CKD-induced expression of collagen (3.2-fold) and the pro-inflammatory chemokines CCL2 (1.8-fold) and CCL3 (2.1-fold) in kidney. Altogether, this study reveals for the first time that chemokines CCL6 and CCL9 are upregulated early in experimental CKD, with CCL9-blockade during CKD initiation enhancing kidney inflammation and fibrosis.

## 1. Introduction

Chronic kidney disease (CKD), with an estimated global prevalence of more than 10% [1], is defined by a decrease in kidney filtration function and/or the presence of kidney damage markers for over 90 days [2]. Based on the estimated glomerular filtration rate (eGFR) and albuminuria together as a measure of filtration capacity, CKD is classified in five stages [1]. In addition to CKD burden, patients with CKD also suffer from increased risk of cardiovascular disease, with CKD identified as an independent risk factor of cardiovascular morbidity and mortality [3,4]. In both diseases, organ inflammation and fibrosis play important pathophysiological roles.

Initial kidney injury triggers the expression of pro-inflammatory mediators as well as the recruitment and accumulation of inflammatory leukocytes, including neutrophils, monocytes and macrophages, in the kidney. These contribute to further organ damage and also produce pro-fibrotic mediators triggering fibrotic phenotypes [5,6,7]. Fibrosis following tissue injury is due to the excessive formation of extracellular matrix proteins, e.g., collagen, and further promotes organ dysfunction [8]. Patients with CKD often exhibit kidney fibrosis, detectable as glomerulosclerosis, arterio- and arteriolosclerosis and/or tubulointerstitial fibrosis, which is associated with an impairment of kidney function [8,9].

Key players in the fibrosis-driving inflammatory response are chemokines and their receptors. They play an important role in leukocyte chemotaxis and associated inflammation, as shown in the context of both cardiovascular disease [10] and CKD [5]. For example, C-C chemokine receptors type 1 and 2 (CCR1, CCR2) expressed by neutrophils and monocytes mediate their infiltration in the injured kidney [11,12,13]. In addition to various chemokine receptors, up to 50 chemokines have been identified, which often can bind multiple receptors [14]. Despite a number of studies, many chemokines currently remain uninvestigated in CKD. Thus, we initiated our study with chemokine profiling to identify chemokines upregulated in an early stage of experimental CKD. This revealed an upregulation of mouse chemokine (C-C motif) ligands 6 and 9 (CCL6, CCL9) in different CKD mouse models, with CCL6 and CCL9 the only two mouse chemokines belonging to the NC6 subfamily of CC-chemokines, a subclass not yet studied in CKD. With the closest related human chemokine CCL15 also upregulated in CKD patients [15], the upregulation of CCL6 and CCL9 detected in the early stage of CKD and the fact that both chemokines have been described as ligands of the pro-inflammatory and pro-fibrotic CCR1 [16], we here investigated a potential role of these chemokines in kidney inflammation and fibrosis in early experimental CKD.

## 2. Materials and Methods

### 2.1. Animal Experiments

All animal experiments were approved by local regulatory authorities and performed according to local and national ethical guidelines. Mice were 8–12 weeks old at experiment start and had free access to food and water. Chemokine screening was performed in 129/Sv mice (mixed gender, purchased from Charles River, Wilmington, NC, USA) subjected to one-step 5/6 nephrectomy, with sham-operated animals as controls. Chemokines were also quantified in male C57BL/6N mice (purchased from Charles River, Wilmington, NC, USA) and C57BL/6J *apolipoprotein E* deficient mice (*ApoE^−/−^*, own breeding) with adenine-induced CKD. C57BL/6N mice were fed a 0.2% adenine-rich diet (Altromin, Lage, Germany) for 3 weeks. C57BL/6J mice received a high-fat diet (HFD: Altromin Western Type diet) for 4 weeks, followed by HFD mixed with 0.3% adenine/19.5% casein for 10 days and HFD with 0.15% adenine/19.5% casein for a further 4 days. Control mice over the last 14 days received the same HFD with 19.5% casein but without adenine.

The role of mouse chemokines CCL6 (Entrez Gene ID 20305; also called C10) and CCL9 (Entrez Gene ID 20308; also called MIP-1γ) was studied in two sets of experiments. In the first set of experiments, female C57BL/6J mice (purchased from Janvier, Le Genest-Saint-Isle, France) were implanted with a mini-osmotic pump (Alzet, Model 2002, Cupertino, CA, USA) for continuous delivery of endotoxin-free mouse CCL6 (amino acids 42–116) or mouse CCL9 (amino acids 50–122) (both from R&D systems, Minneapolis, MN, USA; 1 µg/day; 0.5 µL/h), or with 0.9% NaCl as vehicle control. Pumps were implanted subcutaneously (s.c.) under 1.5–2% isoflurane anesthetics, preceded by 0.1 mg/kg s.c. Buprenorphine treatment. On the day of pump implantation, CKD was induced by feeding a 0.2% adenine diet (Ssniff, Soest, Germany) for 2 weeks. C57BL/6J mice treated with a vehicle through pump implantation and on standard diet (Ssniff, Soest, Germany) served as non-CKD controls. In the second set of experiments, female mice received a high-fat diet (HFD; Altromin Western Type diet) for 4 weeks, followed by HFD mixed with 0.3% adenine for 10 days to induce CKD. On days 3, 5, 7 and 9 after CKD induction, mice were intraperitoneally injected with a blocking antibody against mouse CCL6 (R&D systems, Minneapolis, MN, USA, AF487) or CCL9 (R&D systems, Minneapolis, MN, USA, MAB463), in total 10 µg in 0.9% NaCl per injection. ‘Isotype CKD controls’ were on the same diet but received the isotype-matched antibody (R&D systems, Minneapolis, MN, USA, MAB005). Hyperlipidemic *Apoe^−/−^* mice without adenine but with isotype-matched antibody treatment served as non-CKD controls (‘isotype controls’).

### 2.2. Blood Sampling and Organ Isolation

Upon sacrifice, blood was collected by heart cannulation under anesthetics with ketamine (100 mg/kg) and xylazine (10 mg/kg). Serum was prepared and stored at −80 °C. Serum creatinine and urea were quantified by clinical laboratory routine (Vitros 350, Ortho Clinical Diagnostics, Raritan, NJ, USA). After gentle in vivo rinsing with PBS, kidneys were harvested for flow cytometric analysis, histological tissue analysis or snap-frozen in liquid nitrogen and stored at −80 °C for protein extraction.

### 2.3. Leukocyte Profiling and Flow Cytometry

Leukocyte counts in blood were determined using a Celltac MEK-6550 (Nihon Kohden, Tokyo, Japan), with differential blood counts obtained through Wright’s stain. Half of a kidney was mechanically minced using a scalpel and digested with 0.25 mg/mL Liberase (Merck, Darmstadt, Germany) in RPMI-1640 medium at 37 °C for 1 h. The resulting solution was sieved through a 70 µm cell strainer (Greiner Bio-One, Kremsmünster, Austria) and the enzymatic reaction stopped via diluting the solution in RPMI-1640 with 10% fetal calf serum (medium and supplement from Thermo Fisher Scientific, Waltham, MA, USA). Subsequently, cells were pelleted by centrifugation, washed in HANKS Complete buffer (1x HBSS with 0.3 mM EDTA and 0.1% bovine serum albumin) and stained with antibody mixtures directed against CD115 (Invitrogen, Waltham, MA, USA), CD11b, CD45 (BD Pharmingen, Franklin Lakes, NJ, USA), Ly-6G (Gr1), F4/80 (eBioscience, Santa Clara, CA, USA) and CD206 (BioLegend, San Diego, CA, USA). CountBright^TM^ Absolute Counting Beads (Invitrogen, Waltham, MA, USA) were added for absolute cell counting. Stained cells were analyzed by flow cytometry using a FACSCanto II and FACSDiva software (BD Biosciences, Franklin Lakes, NJ, USA) with appropriate fluorescence compensation. Using FlowJo^TM^ software (for Windows, version 10.7.1, BD Life Science, Ashland, OR, USA), cell populations were gated and analyzed as follows: leukocytes (CD45+), neutrophils (CD45+ CD11b+ CD115- Ly-6G+), monocytes (CD45+ CD11b+ CD115+) with Ly-6G -high and Ly-6G -low subsets, tissue macrophages (CD45+ CD11b+ F4/80+) with subsets M1 (CD206-) or M2 (CD206+).

### 2.4. Histological Tissue Analysis: Acid Fuchsin Orange G (AFOG) Staining

Longitudinally cut kidneys were fixed for 24–48 h in methyl Carnoy’s solution directly after organ isolation. Fixed samples were dehydrated in ascending alcohol concentrations, paraffin embedded and cut into 1 µm sections using a rotation microtome. For histological analyses, the slides were deparaffinized and fixed in Bouin’s solution for 2 h at 60 °C, rehydrated and sequentially treated with hematoxylin and iron chloride solution (1 min), 0.1% hydrochloric acid (10 s), 1% phosphomolybdic acid (5 min) and AFOG solution (10 min), each separated by a washing step in water. Finally, slides were dehydrated and covered with Histokitt for microscopic analysis.

### 2.5. Tissue Protein Isolation, Western Blot Analysis and ELISA

Approximately 5–10 mg of kidney tissue, snap-frozen in liquid nitrogen, was lysed in 150 µL of an ice-cold, non-denaturing lysis buffer (consisting of Cell Lysis Buffer (Cell Signaling, Cambridge, UK), c0mplete^TM^ Mini Protease Inhibitor Cocktail (Roche, Basel, Switzerland) and PhosSTOP (Sigma-Aldrich, St. Louis, MO, USA)), using steel beads for homogenization with a Qiagen tissue lyser (Qiagen, Hilden, Germany). After centrifugation, supernatant was collected, measured for protein concentration using a NanoDrop One (Thermo Fisher Scientific, Waltham, MA, USA) and stored at −80 °C for further analysis.

For Western blot analysis, 15 µg total protein was mixed with 4x Laemmli protein sample buffer (BioRad, Hercules, CA, USA) and heated to 95 °C for 5 min before analysis over SDS-PAGE and standard Western blot detection protocols. Primary antibodies were used against collagen 1 (1310-01, SouthernBiotech, Birmingham, AL, USA) and β-actin (4967, Cell Signaling, Cambridge, UK), with secondary antibodies HRP-conjugated (Santa Cruz Biotechnology, Dallas, TX, USA or Cell Signaling, Cambridge, UK) for chemiluminescent detection (Super Signal^TM^ West Pico PLUS Chemiluminescent Substrate, ThermoFisher Scientific, Waltham, MA, USA) with a GelDoc XR (BioRad, Hercules, CA, USA). Image Lab (Version 2.0, BioRad, Hercules, CA, USA) was used for quantitative analysis by normalizing the band intensities to β-actin as loading control.

CCL6 and CCL9 levels in serum and kidney lysates were quantified by using a commercially available ELISA (DuoSet, R&D Systems, Minneapolis, MN, USA) according to the manufacturer’s instructions.

### 2.6. Chemokine Profiling and LUNARIS Assay

Chemokine profiling in serum of CKD mice was performed using a Proteome Profiler^TM^ Array ‘Mouse Chemokine Array Kit’ (R&D Systems, Minneapolis, MN, USA), according to the manufacturer’s instructions. Levels of the chemokines CCL2 (MCP1), CCL3, CCL4, CCL5 (RANTES), CCL11, CCL19, CCL20, CCL22, CXCL10 (IP10), CXCL11 and CX3CL1 (Fractalkine) in kidney lysates were quantified using a LUNARIS Custom Kit 20103S assay (CUST-20103S, Ayoxxa, Cologne, Germany) according to the manufacturer’s instructions. Analysis of data was performed using LUNARIS Analysis Suite Software (Version 1.4, Ayoxxa, Cologne, Germany).

### 2.7. Statistics

Data are shown as individual values and mean ± SD. All statistical analyses were performed by using GraphPad Prism (Version 9 or higher, GraphPad Software, San Diego, CA, USA). After testing for normality, *p*-values were calculated using student’s *t*-test (unpaired, two-tailed) for two-group comparisons with parametric data, Mann–Whitney test (unpaired, two-tailed) for two-group comparisons with non-parametric data, Kruskal–Wallis test with Dunn’s post-test (non-parametric) or one-way ANOVA (parametric) or two-way ANOVA with Dunnett’s multiple comparisons tests for multiple group comparisons, as appropriate. Outliers were identified with the Grubbs’ test. *p*-value < 0.05 was considered to be statistically significant (* *p* < 0.05, ** *p* < 0.01, *** *p* < 0.001, **** *p* < 0.0001).

## 3. Results

### 3.1. Chemokines CCL6 and CCL9 Are Increased in CKD

**Figure 1:**
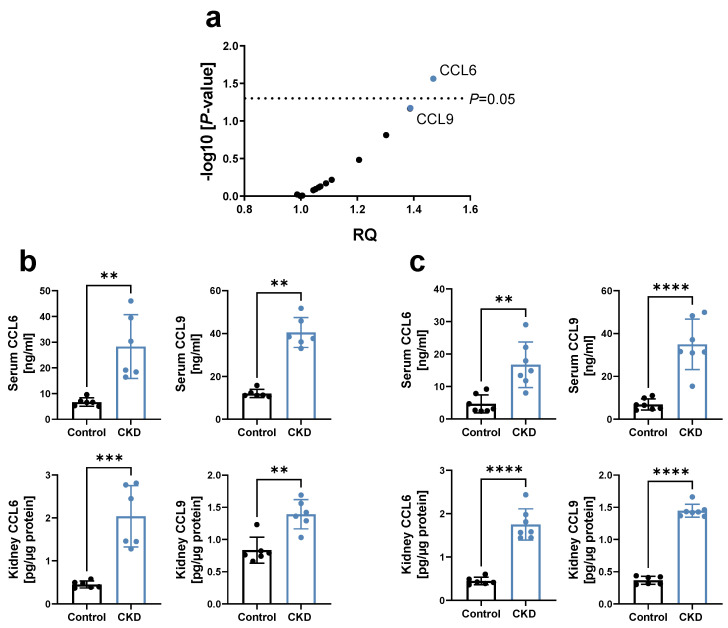
Chemokines CCL6 and CCL9 are increased in experimental CKD. (**a**) Chemokine profiling using a ‘Mouse Chemokine Array Kit’ in serum of 129/Sv mice 3 weeks after 5/6 Nx, relative to sham-operated controls (n = 5–7). RQ = relative quantity, with each dot representing the mean RQ of an analyzed chemokine; CCL6 and CCL9 are highlighted in blue. *p* via unpaired t-tests with single pooled variance. (**b**,**c**) Concentration of CCL6 and CCL9 in serum and kidney lysates of (**b**) C57BL/6N mice after 3 weeks of 0.2% adenine or control diet (n = 6); and (**c**) hyperlipidemic C57BL6/J *ApoE^−/−^* mice fed a HFD for 4 weeks, followed by 2 weeks of a 0.3%/0.15% adenine-HFD compared to HFD diet without adenine (n = 6–7). HFD = high-fat diet. (**b**,**c**) Data represent means ± SD. Unpaired two-tailed *t*-test or Mann–Whitney test, comparing CKD animals vs. controls. ** *p* < 0.01; *** *p* < 0.001; **** *p* < 0.0001.

To identify the effect of CKD on systemic chemokine levels in an early stage, a chemokine profiling was performed in the blood of 129/Sv mice 3 weeks after 5/6 nephrectomy (5/6 Nx). This revealed a significant increase in CCL6, with CCL9 being the chemokine with the second highest upregulation (Figure 1a). CCL6 (also known as C10 or MRP-1) and CCL9 (also known as MIP-1γ or MRP-2) are the only two mouse chemokines belonging to the NC6 subfamily of CC-chemokines, based on a separately encoded N-terminal extension upstream of the chemokine body compared to other CC-chemokines [16,17,18]. We thus further examined the expression of these chemokines in CKD, and revealed that both CCL6 and CCL9 were also upregulated in the early stage of adenine-induced CKD in C57BL/6N mice, in serum as well as kidney lysates (Figure 1b). Similar observations were made in hyperlipidemic C57BL/6J *ApoE^−/−^* mice on a high-fat, adenine-rich diet (Figure 1c), with the latter CKD mouse model chosen based on the high clinical relevance of hyperlipidemia as a risk factor for both CKD [19] and CVD [20].

### 3.2. Blocking CCL9 Increases Kidney Fibrosis during CKD Induction

We hypothesized that in vivo treatment with CCL6 or CCL9 during CKD induction would enhance kidney inflammation. However, continuous delivery of CCL6 or CCL9 to C57BL/6J mice during the 2 weeks of feeding with 0.2% adenine diet almost completely abolished the adenine-induced infiltration of macrophages and overall CD11b^+^ cells in the kidney (* for CCL9; *p* = 0.0772 for CCL6) (Supplementary Figure S1a,b). This was not accompanied by a significant effect on counts of leukocytes, neutrophils, monocytes or lymphocytes in peripheral blood (Supplementary Figure S1c). Our CKD protocol was insufficient to induce kidney dysfunction in terms of serum creatinine or urea changes, and the additional treatment with CCL6 or CCL9 did not impact these markers either in this early stage of CKD induction (data not shown).

**Figure 2:**
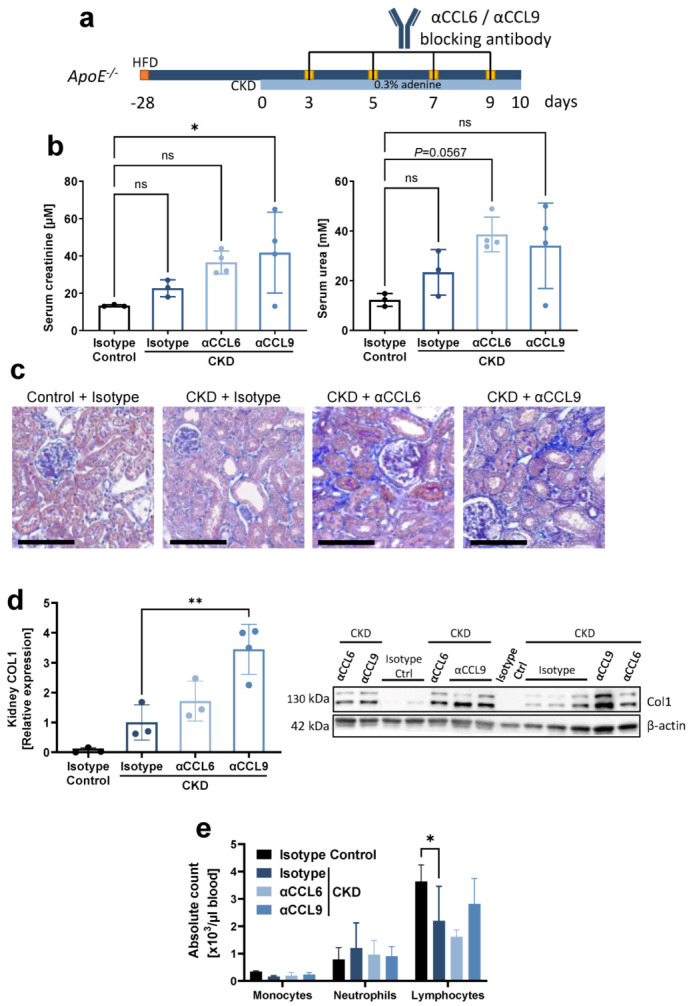
Systemic antibody-mediated blocking of CCL9 increases CKD-induced kidney fibrosis without effect on systemic inflammatory cells. Hyperlipidemic *ApoE^−/−^* mice with adenine-induced CKD were treated with blocking antibodies against CCL6 (αCCL6 CKD) or CCL9 (αCCL9 CKD), or with isotype-matched antibody controls (Isotype CKD), as indicated (n = 3–4). Hyperlipidemic *ApoE^−/−^* mice without adenine but with isotype-matched antibody treatment served as non-CKD controls (Isotype Control). (**a**) Experimental timeline. CKD = chronic kidney disease; HFD = high-fat diet. (**b**) Serum creatinine and urea at the end point. (**c**) Representative images of AFOG staining of kidney sections revealing kidney damage in all CKD conditions. Scale bar = 100 µm. (**d**) Quantification of collagen 1 (COL1) in kidney lysates by Western blot, normalized to β-actin and displayed relative to non-CKD controls. One value for ‘αCCL6 CKD’ excluded due to incomplete blotting (full Western blot images available online). (**e**) Leukocyte cell counts in peripheral blood. (**b**,**d**,**e**) Data represent means ± SD. Kruskal–Wallis test with Dunn’s post-test, one-way ANOVA with Dunnett’s post-test, or two-way ANOVA with Dunnett’s post-test for multiple comparisons, as appropriate. * *p* < 0.05; ** *p* < 0.01; *ns =* not significant.

In a proof of concept experiment, we next examined the effects of blocking the two chemokines during CKD induction in hyperlipidemic C57BL6/J *ApoE^−/−^* mice. More specifically, during 10 days of CKD induction by supplementing a high-fat diet with 0.3% adenine, mice received four i.p. injections with neutralizing antibodies directed against mouse CCL6 or CCL9 (Figure 2a). As controls, mice on the same diet were treated with isotype control antibodies (‘isotype CKD’). *ApoE^−/−^* mice on high-fat diet without adenine but with isotype-matched antibody treatment served as non-CKD isotype controls (Figure 2a). During this early stage of CKD induction, no significant increase in serum creatinine or urea was observed when comparing isotype CKD controls with non-CKD mice. However, serum creatinine significantly increased when blocking CCL9 during CKD induction (Figure 2b). Histology confirmed early kidney damage with initial kidney fibrosis upon adenine feeding (Figure 2c). Furthermore, kidney fibrosis significantly increased in αCCL9-treated CKD mice compared to isotype-treated CKD controls, as revealed by quantification of collagen 1 in kidney protein lysates (Figure 2d). Blocking CCL6 or CCL9 did not significantly alter the absolute numbers of monocytes, neutrophils or lymphocytes in the blood (Figure 2e). Overall, and in contrast to our initial hypothesis, these findings unexpectedly revealed that CCL9 blockade increases CKD-induced kidney fibrosis without effect on systemic inflammatory cells.

### 3.3. Blocking CCL9 Enhances Kidney Inflammation during CKD Induction

**Figure 3:**
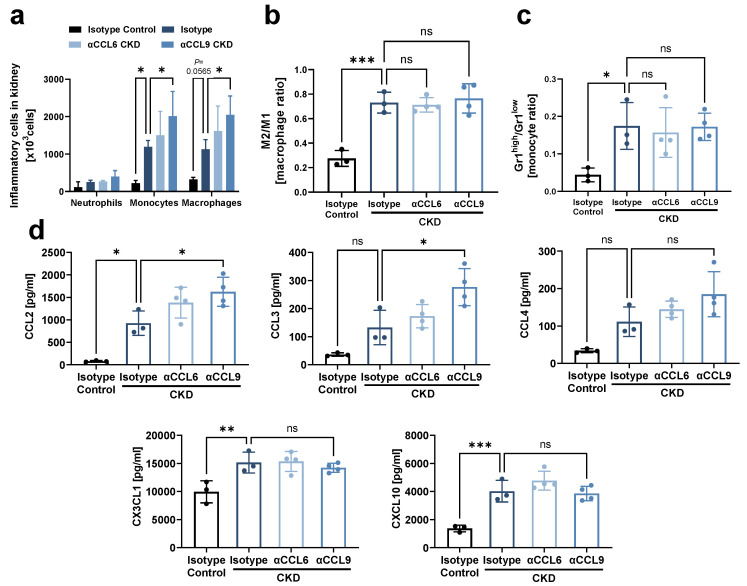
Systemic antibody-mediated blocking of CCL9 increases kidney inflammation. As in Figure 2a, hyperlipidemic *ApoE^−/−^* mice with adenine-induced CKD were treated with blocking antibodies against CCL6 (αCCL6 CKD) or CCL9 (αCCL9 CKD), or with isotype-matched antibody controls (Isotype CKD) (n = 3–4). Hyperlipidemic *ApoE^−/−^* mice without adenine but with isotype-matched antibody treatment served as non-CKD controls (Isotype Control). (**a**) Flow cytometric analysis of neutrophils, monocytes and macrophages in kidney. (**b**,**c**) The ratio of (**b**) M2 vs. M1 macrophages and (**c**) Ly-6G^high^ vs. Ly-6G^low^ monocytes in kidney by flow cytometric analysis. (**d**) Chemokine concentration in kidney analyzed using a LUNARIS assay. (**a**–**d**) Two-way ANOVA (**a**) or one-way ANOVA (**b**–**d**) with Dunnett’s post-test for multiple comparisons. * *p* < 0.05, ** *p* < 0.01, *** *p* < 0.001, *ns =* not significant.

Since inflammation and fibrosis are highly interlinked in the damaged kidney [19], we next analyzed the effect of chemokine blockade on inflammatory processes in the kidney during CKD development. Flow cytometric analysis revealed that monocytes and macrophages increasingly accumulated in the kidney during CKD induction, and this was further aggravated by blocking CCL9 as compared to isotype CKD controls (Figure 3a, Supplementary Figure S2a,b). Of note, although CKD induction favored the accumulation of M2 over M1 macrophages and increased the ratio of Ly-6G^high^ over Ly-6G^low^ monocytes compared to non-CKD controls, these ratios were not significantly altered by blocking of either CCL6 or CCL9 (Figure 3b,c). Finally, CKD induction created a pro-inflammatory milieu in the kidney with enhanced levels of the pro-inflammatory chemokines CCL2, CX3CL1 and CXCL10, as revealed by a chemokine LUNARIS assay. CCL9 blockade further increased kidney levels of CCL2 as well as of the pro-inflammatory chemokine CCL3. No impact on CKD-induced upregulation of CX3CL1, CXCL10 and CCL20 was found (Figure 3d, Supplementary Figure S3). CCL22 was not induced by CKD, whereas levels of CCL5, CCL11, CCL19 and CXCL1 were outside of the quantitative range (data not shown). Taken together, these data indicate that CCL9 blockade enhances kidney inflammation during CKD induction.

## 4. Discussion

Early infiltration of pro-inflammatory leukocytes in the damaged kidney contributes to kidney fibrosis and CKD progression [7]. In this study, we identified an early upregulation of the chemokines CCL6 and CCL9 in experimental CKD, with CCL9 blockade during CKD initiation unexpectedly enhancing kidney inflammation and fibrosis.

Initial kidney injury triggers the expression of pro-inflammatory mediators, including cytokines and chemokines that upregulate inflammatory processes and mediate the recruitment of inflammatory leukocytes. These contribute to further organ damage and also produce pro-fibrotic mediators triggering fibrotic phenotypes [5,6]. For example, the chemokine CCL2 was upregulated already 3 hours after induction of glomerulonephritis by an anti-thymocyte antibody in rats, and anti-CCL2 antiserum reduced neutrophil and monocyte/macrophage accumulation in the injured kidney [21,22]. In patients with CKD, interstitial macrophage numbers in kidney biopsies are closely correlated with kidney damage, with urinary CCL2 levels and interstitial macrophage numbers interdependent parameters in multivariate analysis [23]. The chemokine ligands CCL3 and CCL5, both agonists of the CCR1 receptor, were significantly upregulated in the progressively injured kidney, as revealed after 6 weeks of adriamycin-induced nephropathy [24]. Subsequent studies showed that the CCR1 receptor contributes to kidney fibrosis through the recruitment and accumulation of macrophages in two models of kidney diseases [11,24].

We initiated our study with a chemokine profiling in blood 3 weeks after 5/6 nephrectomy (5/6 Nx) to identify chemokine dysregulation in early stages of CKD, i.e., being potentially the initial steps in the disease pathogenesis. CCL6 and CCL9 were the highest upregulated mediators and also increased in two other mouse models of adenine-induced nephropathy. Both chemokines are mostly related to human CCL15 and CCL23 [16], with CCL15 recently identified to be upregulated in CKD patients [14]. As for human CCL15 and CCL23, mouse CCL6 and CCL9 have been described as agonists of the chemokine receptor CCR1 [16,25,26].

Given that no data were available on the role of both upregulated chemokines in CKD, we examined the effect of chemokine treatment and blockade. Unexpectedly, macrophage and monocyte accumulation was reduced by chemokine treatment, whereas it was aggravated by blockade of CCL9, although without significant impact on the ratio of M2 to M1 macrophages. Monocytes and pro-inflammatory M1 macrophages are well-known to contribute to ongoing kidney inflammation and damage [27]. Moreover, although M2 macrophages can mediate anti-inflammatory effects upon acute kidney injury, they contribute to CKD by promoting kidney fibrosis and increasingly accumulate in the injured kidney during CKD development [27,28,29,30,31]. In line with the increased accumulation of both M1 and M2 macrophages in kidney upon CCL9 blockade during CKD onset, these animals displayed a higher pro-inflammatory milieu (with increased levels of CCL2 and CCL3) as well as increased kidney fibrosis upon CKD induction. CCL2, CCL3 and CCL5 (the latter remaining outside of the quantitative range of our assay) are agonists of the chemokine receptors CCR2 (for CCL2) and CCR1 (for CCL3, CCL5), which are expressed on macrophages. CCL2 [21,22,32] and its receptor CCR2 [13] as well as CCL5 [32] and its receptor CCR1 [11,33] play an important role in macrophage infiltration in the damaged kidney, as evident from multiple experimental studies. Upregulation of CCL2 and CCL5, and to a lesser extent CCL3, was also shown to mediate the accumulation of macrophages in kidney in a mouse model of the hemolytic-uremic syndrome [34]. These findings jointly reveal especially CCL2, but also CCL5 and CCL3, as important drivers of inflammatory macrophage accumulation in the injured kidney. Both tubular epithelial cells and infiltrating leukocytes contribute to the production of CCL2, CCL3 and CCL5 in injured kidneys [12,13,35,36,37], and mice lacking CCR1 also show reduced upregulation of the CCR1 ligands CCL3 and CCL5 in kidney along with reduced neutrophil and macrophage infiltration upon kidney injury [12]. Combined, these findings suggest a positive feedback loop between increased pro-inflammatory leukocyte infiltration and enhanced production of pro-inflammatory CCL2 and CCL3, as also observed in our CKD study with CCL9 blockade.

Furthermore, this positive feedback loop with increased CCL2 and CCL3 production upon CCL9 blockade may also further promote kidney fibrosis, as shown for both CCL2 and CCL3. Indeed, antibody-mediated neutralization of CCL2 was previously shown to reduce the development of kidney fibrosis in nephrotoxic serum-induced glomerulonephritis [32]. Furthermore, deficiency of the CCL2 receptor CCR2, and to a lesser extent deficiency of CCL3, reduced kidney fibrosis after unilateral ureter obstruction (UUO) [13]. Thus, with CCL2, CCL3 as well as M2 macrophages known to be crucial drivers of kidney fibrosis, the increased accumulation of these chemokines and M2 macrophages in kidney upon CCL9 blockade during CKD onset could further explain the observed parallel increase in kidney fibrosis in our study.

The initial trigger that links CCL9 antibody-mediated neutralization to either macrophage accumulation or increased CCL2/CCL3 production currently remains unclear. Since CCL9 has been described as a ligand of CCR1 [16], CCL9 neutralization in vivo might favor CCR1 binding to its ligands CCL3 and CCL5, and in this way trigger increased kidney inflammation and subsequently fibrosis [5,11]. Additionally, interference with CCL9 may alleviate the constraining effect of CCL9 on the pro-inflammatory and pro-fibrotic effect of CCL2/CCR2 signaling, as was also revealed for *Ccl5*-knockout. Indeed, in a model of hypertensive kidney injury, *Ccl5*-knockout surprisingly enhanced macrophage infiltration, levels of CCL2 (but not CCL3) and the pro-inflammatory cytokines IL1β and TNF, as well as fibrosis in the kidney, with comparable observations upon UUO [38]. Since *Ccl5*-knockout did not induce any significant effects on UUO-induced kidney fibrosis or inflammation in the presence of CCL2 blockade, upregulated CCL2 levels seemed to underlie increased kidney damage upon *Ccl5*-deficiency [38]. As another potential mechanism, CCL9 might be competing for glycosaminoglycan (GAG) binding with pro-inflammatory and/or pro-fibrotic chemokines or other factors. Chemokine binding to GAGs on the endothelium as well as tissue extracellular matrix is important for leukocyte extravasation and thereby tissue accumulation [39]. Accordingly, interference with chemokine-GAG interactions, for example by using peptides with high GAG affinity, has been suggested as a potential strategy to target chemokines and their roles in leukocyte recruitment and inflammation [39]. Recently, in a contact sensitivity model, a GAG-binding C-terminal fragment of the CXCL9 chemokine was shown to prevent locally produced chemokines from recruiting leukocytes by competing for GAG binding [40]. Moreover, chemokine CXCL10 has been shown to act anti-fibrotic in the lung through interaction with syndecan-4, a proteoglycan with covalently attached GAG chains. This anti-fibrotic effect occurred independently of its receptor CXCR3, most likely by competition with pro-fibrotic factors for GAG binding [41]. Whether similar mechanisms underlie pro-inflammatory and pro-fibrotic effects of CCL9 neutralization, with CCL9 competing for either chemokine receptor or GAG binding with pro-inflammatory/pro-fibrotic chemokines or other factors, currently remains unclear. Of note, opposite to increased macrophage accumulation in kidney upon CCL9 neutralization, CCL9 peptide treatment significantly counteracted macrophage accumulation upon CKD induction, thus confirming a role for CCL9 in blocking kidney inflammation in CKD.

Finally, CCL6 peptide treatment during CKD induction also highly reduced inflammatory cell infiltration in kidney. Nonetheless, compared to CCL9 neutralization, blockade of CCL6 did not increase the overall kidney collagen content in CKD mice and also had less impact on inflammatory cell infiltration and chemokine production in the kidneys of these mice. Whether this is caused by a compensatory role of CCL9 (or other upregulated chemokines) and whether CCL6 blockade could still provide an additive effect on kidney inflammation and fibrosis in CKD in the absence or neutralization of CCL9, currently remains unclear.

## 5. Conclusions

In summary, in this study, we provided first insights into the role of CCL9 in CKD, with CCL9 upregulated early in CKD and counteracting kidney inflammation and fibrosis. Whether the human homologue CCL15 could exert comparable effects or would be a useful biomarker of kidney inflammation and fibrosis, remains to be investigated in future studies.

## Data Availability

All data presented in this study are available upon reasonable request from the corresponding author.

## Supplementary Materials

The following are available online at https://www.mdpi.com/article/10.3390/biomedicines10020420/s1, Figure S1: CCL9 treatment reduces kidney inflammation in CKD, Figure S2: Flow cytometry gating of leukocyte subsets in kidney, Figure S3: Systemic antibody-mediated blocking of CCL9 or CCL6 does not affect kidney CCL20 expression in CKD. In addition, complete Western blot images are available as Online Supplemental Information.
