# Supplementary material for: Chemokine CCL9 Is Upregulated Early in Chronic Kidney Disease and Counteracts Kidney Inflammation and Fibrosis
Source: Biomedicines. 2022 Feb 10;10(2):420. doi: 10.3390/biomedicines10020420 (PMC8962359; doi:10.3390/biomedicines10020420)
Supplement: Supplementary file 1 [file biomedicines-10-00420-s001.zip › biomedicines-1591363-supplementary.pdf]

# Chemokine CCL9 is upregulated early in chronic kidney disease and counteracts kidney inflammation and fibrosis

## Supplementary Figures

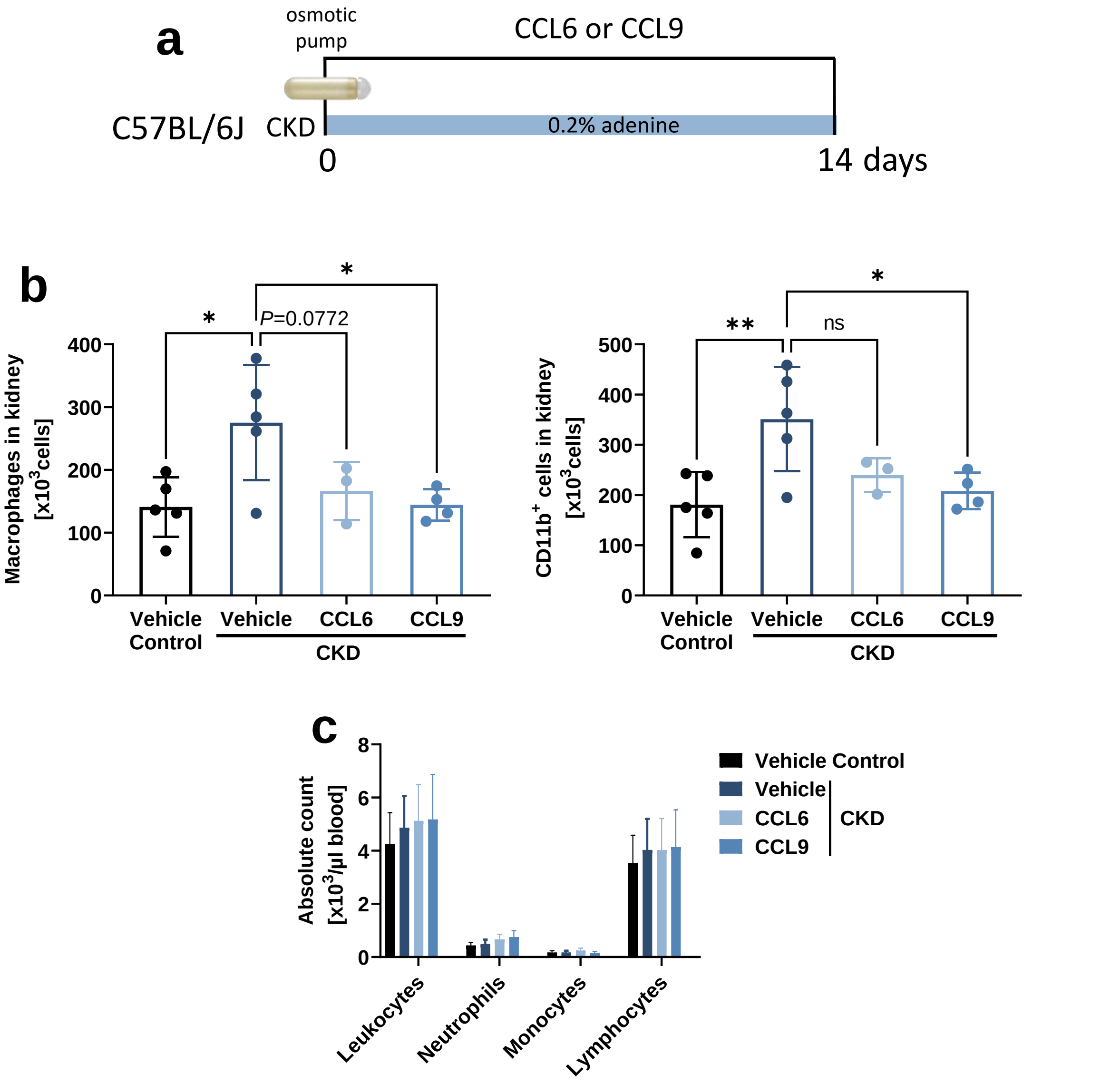


**Figure S1. CCL9 treatment reduces kidney inflammation in CKD.** C57BL/6J fed with 0.2% adenine-diet for two weeks received CCL6 or CCL9, or 0.9% NaCl as vehicle control via osmotic pumps, as indicated (n=3-5). C57BL/6J mice treated with vehicle through pump implantation and on standard diet served as non-CKD controls. **(a)** Experimental timeline. *CKD = chronic kidney disease*. **(b)** Macrophage and CD11b$^+$ cell counts in kidney. **(c)** Leukocyte, neutrophil, monocyte and lymphocyte cell counts in peripheral blood. **(b-c)** Data represent means ± SD. One-way ANOVA with Dunnett's post-test for multiple comparisons, as appropriate. **P*<0.05; ***P*<0.01; *ns = not significant*.

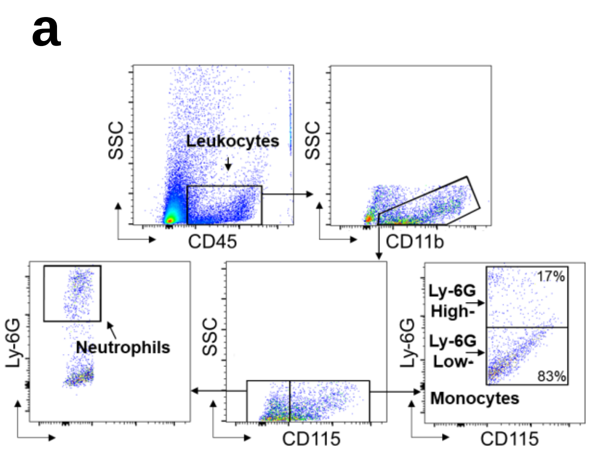


**Figure S2. Flow cytometry gating of leukocyte subsets in kidney. (a)** Gating of neutrophils (CD45+ CD11b+ CD115- Ly-6G+) and monocytes (CD45+ CD11b+ CD115+) with Ly-6G$^{high}$ and Ly-6G$^{low}$ monocyte subsets in kidneys. **(b)** Gating of macrophages (CD45+ CD11b+ F4/80+) with subsets M1 (CD206-) or M2 (CD206+) in kidneys. **(a-b)** Representative images are shown (selected from the CKD + Isotype group).

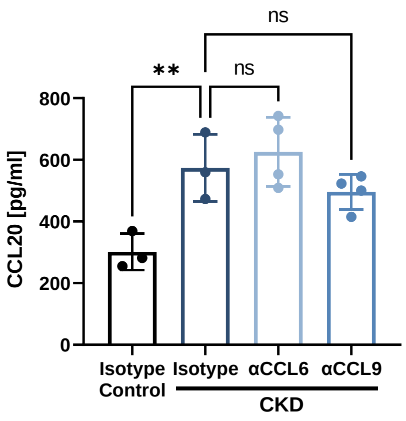


**Figure S3. Systemic antibody-mediated blocking of CCL9 or CCL6 does not affect kidney CCL20 expression in CKD.** As in Figure 2a, hyperlipidemic *ApoE*$^{-/-}$ mice on adenine-induced CKD were treated with blocking antibodies against CCL6 (αCCL6 CKD) or CCL9 (αCCL9 CKD), or with isotype-matched antibody controls (Isotype CKD) (n=3-4). Hyperlipidemic *ApoE*$^{-/-}$ mice without adenine but with isotype-matched antibody treatment served as non-CKD controls (Isotype Vontrol). Chemokine concentration of CCL20 in kidney was analyzed using a LUNARIS assay. One-way ANOVA (b-d) with Dunnett's post-test for multiple comparisons. ns = not significant; ***P*<0.01.
